# Recombinant Expression and Purification of T4 Phage Hoc, Soc, gp23, gp24 Proteins in Native Conformations with Stability Studies

**DOI:** 10.1371/journal.pone.0038902

**Published:** 2012-07-13

**Authors:** Paulina Miernikiewicz, Barbara Owczarek, Agnieszka Piotrowicz, Barbara Boczkowska, Kamila Rzewucka, Grzegorz Figura, Andrey Letarov, Eugene Kulikov, Agnieszka Kopciuch, Kinga Świtała-Jeleń, Anna Oślizło, Katarzyna Hodyra, Jerzy Gubernator, Krystyna Dąbrowska

**Affiliations:** 1 Institute of Immunology and Experimental Therapy, Polish Academy of Sciences, Wroclaw, Poland; 2 Winogradsky Institute of Microbiology, Russian Academy of Sciences, Moscow, Russia; 3 Department of Lipids and Liposomes, Faculty of Biotechnology, University of Wroclaw, Wroclaw, Poland; Instituto Butantan, Brazil

## Abstract

Understanding the biological activity of bacteriophage particles is essential for rational design of bacteriophages with defined pharmacokinetic parameters and to identify the mechanisms of immunobiological activities demonstrated for some bacteriophages. This work requires highly purified preparations of the individual phage structural proteins, possessing native conformation that is essential for their reactivity, and free of incompatible biologically active substances such as bacterial lipopolysaccharide (LPS). In this study we describe expression in *E. coli* and purification of four proteins forming the surface of the bacteriophage T4 head: gp23, gp24, gphoc and gpsoc. We optimized protein expression using a set of chaperones for effective production of soluble proteins in their native conformations. The assistance of chaperones was critical for production of soluble gp23 (chaperone gp31 of T4 phage) and of gpsoc (chaperone TF of *E. coli*). Phage head proteins were purified in native conditions by affinity chromatography and size-exclusion chromatography. Two-step LPS removal allowed immunological purity grade with the average endotoxin activity less than 1 unit per ml of protein preparation. The secondary structure and stability of the proteins were studied using circular dichroism (CD) spectrometry, which confirmed that highly purified proteins preserve their native conformations. In increasing concentration of a denaturant (guanidine hydrochloride), protein stability was proved to increase as follows: gpsoc, gp23, gphoc. The denaturation profile of gp24 protein showed independent domain unfolding with the most stable larger domain. The native purified recombinant phage proteins obtained in this work were shown to be suitable for immunological experiments in vivo and in vitro.

## Introduction

Expanding knowledge and understanding of the substantial role of phages in the biosphere, as well as the potential of phage medical applications, renewed the interest of the Western scientific community in phages [1]–[4]. Practical applications of bacteriophages as antibacterial agents or for potential correction of the composition of natural body-associated bacterial communities [5] will require defined pharmacokinetic parameters and identification of the mechanisms of immunobiological activities demonstrated for some bacteriophages.

The interactions of bacteriophages with human and animal organisms depend on the rate of the elimination of the phages by the immune system. Wild type bacteriophages are rapidly sequestered in the spleen and liver and then degraded by macrophages and other mechanisms [6]–[8]. The time of the circulation of bacteriophages in blood can be significantly changed by modifications in the surface proteins of the viral particles [9], [10]. The interaction of the bacteriophage particles with immune system cells (and probably with other cells of the organism) was recently shown to mediate non-bactericidal biological activities of the phage preparations. Bacteriophage T4 and some of its mutants were shown to exert various effects on mammalian cells and immunity both *in vivo* and *in vitro* [10]–[12].

Investigation of the exact molecular mechanisms of observed phage immunobiological activities requires experimental study of the interaction of surface-exposed individual viral proteins with immune cells and receptor molecules. A wide choice of expression systems exists nowadays, allowing one to produce almost any recombinant proteins in a variety of conditions. The simplest and cheapest heterologous bacterial expression systems are based on *E. coli*, a natural host for T4 bacteriophage. Recombinant proteins produced in *E. coli* always contain a massive amount of bacterial lipopolysaccharide (LPS) and other bacterial products, e.g. bacterial DNA and peptidoglycan. The immune system is highly sensitive to stimulation by microbial-derived substances [13], [14]. Particularly LPS is a potent activator of many physiological processes in animals, both regular and pathological ones, *in vivo* and *in vitro* [15]. Presence of LPS and other immunogenic bacterial components may strongly interfere with investigation of protein activities. Here we present an optimized method for production of gp23, gp24, gphoc and gpsoc proteins, forming the outer surface of the T4 phage head.

The lattice of the T4 head is made of 930 Major Capsid Proteins (gp23) that form 155 hexamers. The centre of each hexamer is occupied by Highly Immunogenic Outer Capsid Protein (gphoc), i.e. 155 molecules per capsid. The gphoc molecule extends about 6 nm away from the head surface. Gphoc has the shape of a dumbbell with a globular head, a neck, and a base that binds to the gp23 hexamers. Eleven head verticles are occupied by pentamers of Head Vertex Protein (gp24), and the twelfth is connected to the tail (via gp20 connector protein). Between gp23 hexamers, a planar mesh of Small Outer Capsid Protein (gpsoc) is incorporated. As in many other phages, T4 head-decorating proteins Hoc and Soc do not cause any changes in the Major Capsid Protein arrangement, and their presence is not essential for phage viability [16]–[19]. These two proteins have been successfully applied in phage display of foreign proteins and peptides on T4 capsid, mostly as novel vaccine platforms but also for phage purification [20], [21]. Gphoc and gpsoc have also been shown to influence T4 phage susceptibility to cellular uptake [22].

We developed procedures that allow us to obtain highly purified native phage proteins suitable for immunological assays both *in vivo* and *in vitro*. Furthermore, we analysed the secondary structure of these proteins by circular dichroism spectroscopy. We also determined the proteins' stability in increasing concentrations of guanidine chloride.

## Results

### Protein expression and purification

**Figure 1:**
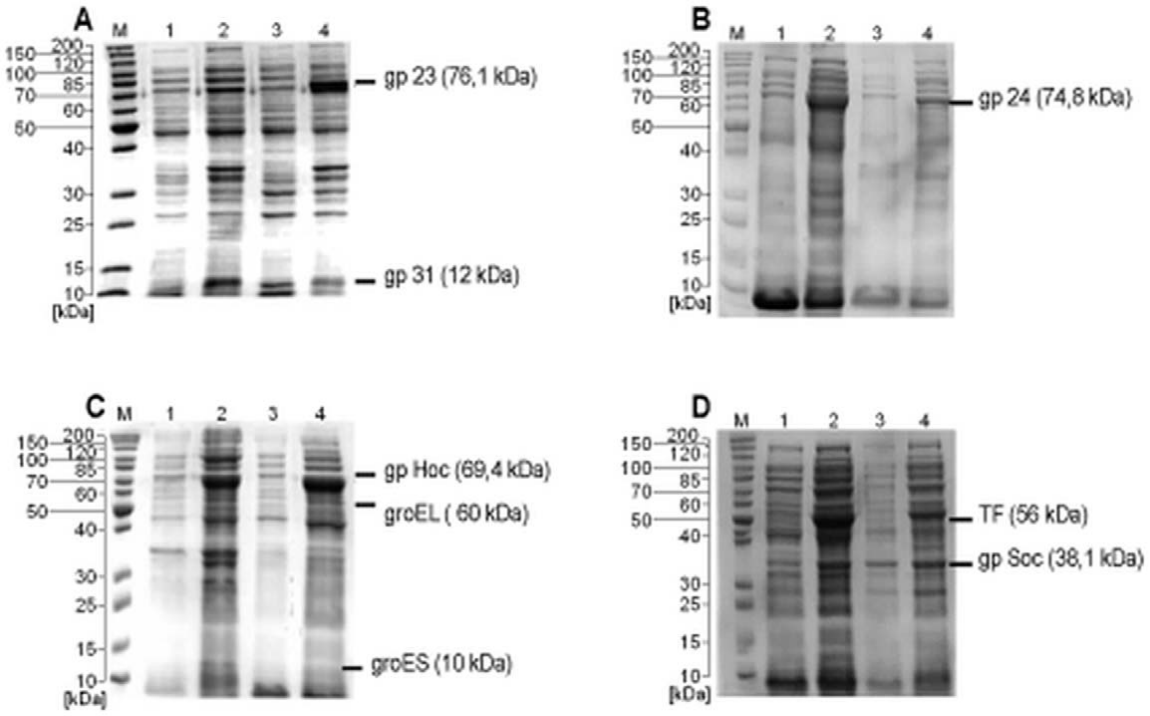
SDS-PAGE of expressed recombinant GST-tagged proteins of T4 phage head. (A) gp23, (B) gp24, (C) gphoc and (D) gpsoc. Overexpressed proteins are marked with dashes. M- molecular weight marker. 1- insoluble fraction of the culture before induction (control). 2- insoluble fraction of the culture after induction (expression). 3- soluble fraction of the culture before induction (control). 4- soluble fraction of the culture after induction (expression).

T4 phage head proteins are not toxic to *Escherichia coli*; therefore they may be easily expressed in the *E. coli* system. *E. coli* is a natural host for T4 bacteriophage and its cytoplasm is a natural environment for T4 protein synthesis. However, massive overexpression of one particular protein usually requires optimization of the expression conditions. Recombinant proteins 23, 24 and Hoc were effectively expressed as N-terminal GST fusions in all tested expression strains, with total yield ranging from 10 to 20 mg per litre of the bacterial culture. However, the yield of the protein Soc when expressed with the N-terminal affinity tag was estimated at less than 0.01 mg in total per litre of the bacterial culture in all tested expression strains and conditions. Therefore this protein was cloned into the pDEST24 vector that provides C-terminal GST. In this case gpsoc yield was 5 to 10 mg (Fig. 1).

In standard expression strains (without chaperones), gp23 and gpsoc were produced as inclusion bodies (Table 1) and were presumably misfolded. Co-expression with a variety of chaperones was used to improve the solubility of these proteins. Folding and solubility of gp23 were substantially improved when this protein was co-expressed with gp31 (a specific chaperone from T4 phage genome) or with Cpn10 and Cpn60 derived from *Oleispira antarctica*, which allow slow expression at 10°C. Gpsoc was expressed mostly as its insoluble form, but the use of TF chaperone allowed this protein to be obtained in sufficient amounts for further isolation in native conditions. Co-expression with chaperones had little effect on the solubility of gp24 and gphoc (Table 1).

**Table 1:** Relative yields of soluble recombinant proteins after co-expression with selected chaperones.

Protein	without chaperones	TF	groEL, groES	dnaK, dnaJ, grpE	gp 31	Cpn10 and Cpn60 (10°C culture)
gp 23	<10%	<10%	<10%	<10%	50–60%	45–55%
gp 24	50–60%	50–60%	50–60%	50–60%	ND*	50–60%
gp Hoc	60–70%	60–70%	70–80%	60–70%	ND*	50–60%
gp Soc	<10%	30–40%	<10%	<10%	ND*	<10%

* ND- not determined.

Relative yields of soluble recombinant proteins after co-expression with selected chaperones, estimated as a fraction of the total amount of the proteins. Parameters representing combinations used in further work were underlined.

**Figure 2:**
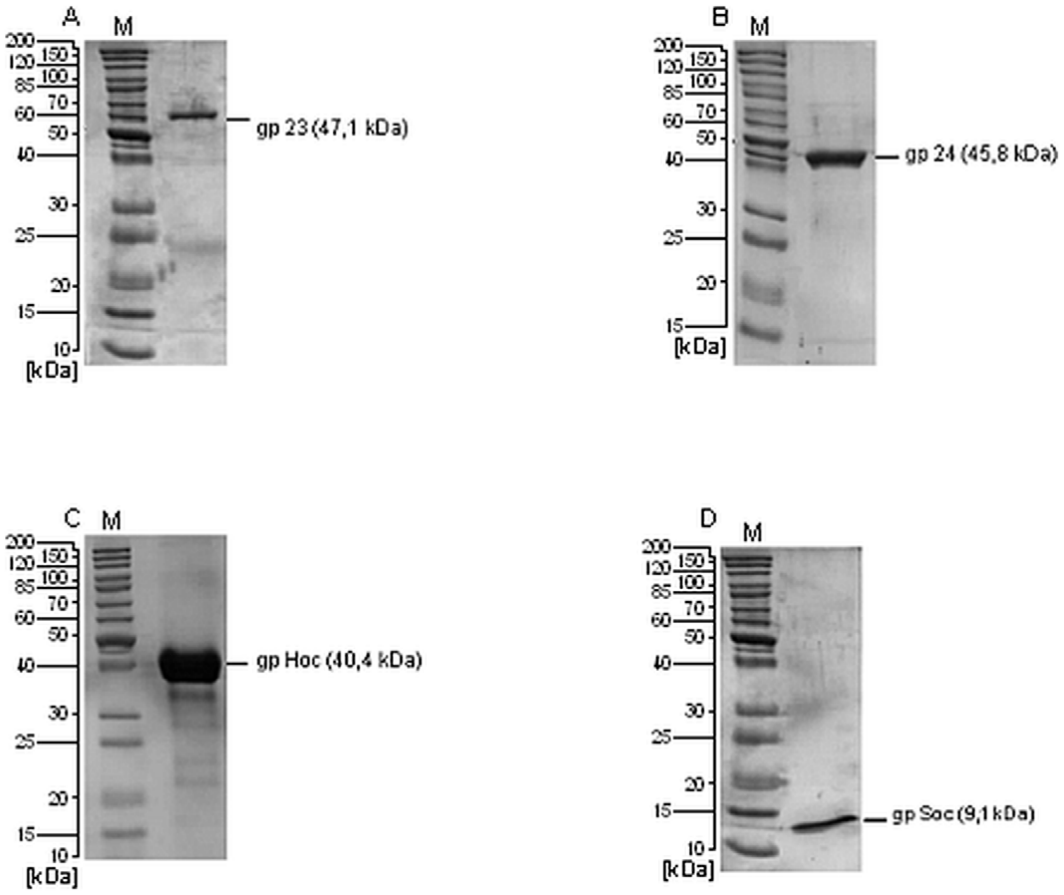
SDS-PAGE of purified proteins of T4 phage head. (A) gp23, (B) gp24, (C) gphoc and (D) gpsoc. Proteins are marked with dashes. M- molecular weight marker.

Proteins were isolated by affinity chromatography, and then the affinity tags were proteolytically cleaved. Subsequent purification was achieved by size exclusion chromatography and by removal of LPS using EndoTrap resin. Immunological purity grade was referred to 10 µg/ml as a typical concentration for immunological assays. Average LPS activity was less than 1 unit per 10 µg of the proteins (Fig. 2, Table 2).

**Table 2:** Endotoxin activity in the phage head protein preparations.

Protein	Minimum	Maximum	Mean
gp 23	0.056	1.157	**0.536**
gp 24	0.014	0.175	**0.100**
gp Hoc	0.007	1.281	**0.404**
gp Soc	0.007	0.737	**0.204**

Mean, maximum and minimum values of six independent preparations were presented (LPS units per 10 microgram of protein preparation).

### Structural analysis of recombinant T4 proteins

**Figure 3:**
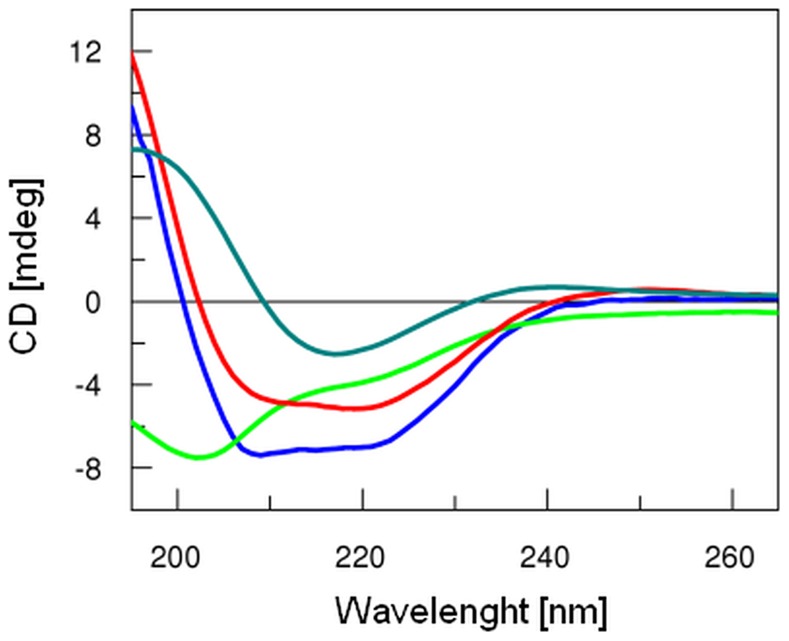
CD spectra of recombinant T4 major head proteins. Comparison of CD spectra acquired in the range of 190–265 nm for recombinant gphoc (dark green), gp24 (blue), gp23 (red), and gpsoc (light green). Analysis of CD spectra showed that produced proteins are folded and contain secondary structures as predicted based on their primary structure (unpublished data).

Heterologous expression and intensive purification procedures may cause the loss of proteins' native structure. Proteins that are soluble but misfolded or locked in the molten globular form are often hard to distinguish from native conformation by a simple test. Purified recombinant proteins, especially structural virion proteins, may also be less stable and accommodate partially unfolded conformations when deprived of their normal molecular surroundings, which can, in turn, alter their immunological properties. As the studied capsid proteins have no activity easy to monitor, their native state and stability have been accessed by measurement of CD spectra. The obtained native spectra were evaluated and secondary structure content was calculated by K2D3 software (www.ogic.ca/projects/k2d3/) (Table 3). All expressed and purified proteins had spectra characteristics for correctly folded proteins (Fig. 3).

**Table 3:** Secondary structure content and thermodynamic parameters of T4 head proteins.

			Secondary structure content		Thermodynamic parameters	
Protein	MW [kDa]	aa	α	β	GdnHCl ½ [M]	ΔG [J/mol]
gp 23	47.1	443	23.3%	26.0%	1.16	5118.68
gp 24	45.8	417	34.1%	15.3%	0.133 (A), 2.42 (B)	575.83 (A), 5301.15 (B)
gp Hoc	40.4	376	1.9%	42.1%	2.03	3628.25
gp Soc	9.1	80	11.0%	16.1%	0.66	559.5

A: domain A; B: domain B.

**Figure 4:**
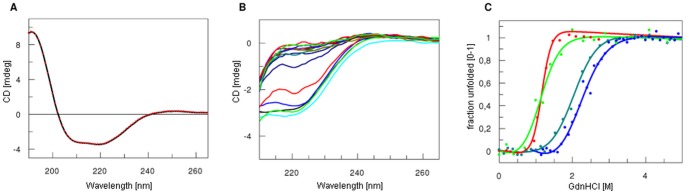
CD spectra and unfolding transitions of the four major T4 capsid proteins. (A) (B) CD spectrum and representative set of chemical denaturation spectra (based on gp23 unfolding induced by increasing concentrations of GdmCl) monitored by changes in ellipticity. (C) Normalized chemical denaturation curves of the gphoc (dark green), gp24 (blue), gp23 (red), and gpsoc (light green); the wave length: 220 nm. The data were analysed assuming a two-state reversible equilibrium transition (data summarized in Table 3).

**Figure 5:**
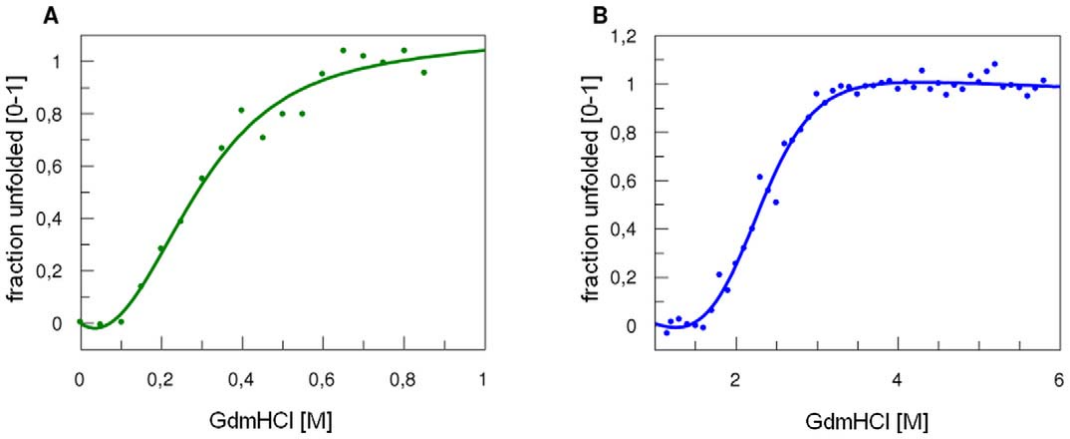
Normalized chemical denaturation curves of the gp24. Domain A (green) and domain B (blue).

**Figure 6:**
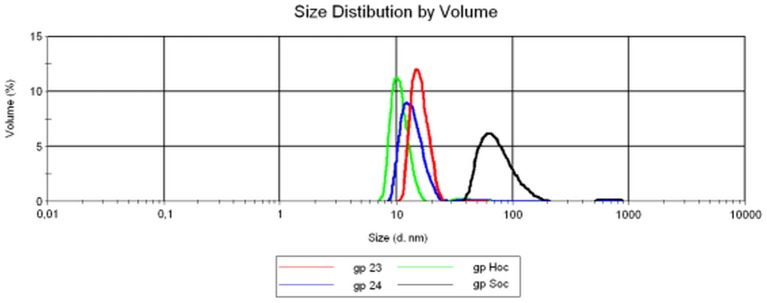
Size distribution in purified preparations of non-denatured T4 head proteins. Dynamic Light Scattering patterns of the native gphoc (green), gp24 (blue), gp23 (red), and gpsoc (black).

Protein stability was studied by measuring changes in the CD signal in increasing denaturant (GdnHCl) concentrations. In these conditions, proteins with defined secondary structure are expected to change their spectra dramatically, from a typical alpha-helical and/or beta-sheet signal to that of a random coil [23], [24]. All investigated proteins showed clear denaturation curves (Fig. 4) and good stability, which makes them suitable for subsequent biological studies. Table 3 summarizes GdnCl½ and deltaG values determined for all the recombinant proteins at pH 7.4 in phosphate buffer. Stability increased as follows: gpsoc, gp23, gphoc. The denaturation profile of gp24 protein showed independent domain unfolding, since the denaturation curve of gp24 protein was bi-modal [25]. The data were analysed separately assuming two-state reversible equilibrium transition for domain A and B respectively (data summarized in Table 3, Fig. 5). A possible effect of protein oligomerization for this bimodal curve of denaturation was excluded by dynamic light scattering, which proved the monomeric nature of gp24 (Fig. 6). Proteins gp23 and gphoc were also monomeric; gpsoc, although soluble and characterized with clear CD spectrum, visibly formed large aggregates (Fig. 6).

## Discussion

In this work we optimized expression and purification of four bacteriophage T4 head proteins in *E. coli*, and obtained their immunological purity grade preparations.

As was known before [26], the major head protein gp23 requires assistance of the phage-encoded chaperonin gp31 that is a functional analogue of bacterial GroES protein able to functionally replace it in interaction with the cellular chaperonin GroEL [27]. However, in our experiments this protein was also produced in native conformation using low temperature expression in presence of Cpn10 and Cpn60 chaperones derived from *Oleispira antarctica* showing 74 and 54% amino acid identity with groEL and groES of *E. coli* (Agilent Technologies, ArcticExpress Specification). Recently, the ability of groEL-groES complex to interact with gp23 was reported [27]. Low temperature expression without chaperones yielded an insoluble product, which leads to the suggestion that Cpn10–Cpn60 complex may possess some feature(s) of GroEL-gp31 complex or groEL–groES complex (possibly the size of internal cavity of the chaperonin) allowing it to assist gp23 folding in T4 infected cells. However, from the practical point of view the best way to produce native gp23 is its co-expression with gp31 protein naturally assisting its folding.

Unexpectedly, effective production of gpsoc, the smallest protein in the T4 head, was difficult due to its poor yield when N-terminal fusion of the affinity tag was used. It is possible that some specific sequences present at the gpsoc C-terminus directs this protein to degradation by bacterial proteases [28]. Therefore the *soc* gene was re-cloned into the expression vector that allowed its expression with a C-terminal affinity tag, resulting in good yield of the protein, but it was almost insoluble. Enhanced expression of chaperone TF improved the solubility to a sufficient level. This result raises a question on the folding of this protein in natural T4 infection. The yield and solubility of gp24 and gphoc were not substantially affected by any tested non-specific chaperones.

The optimised purification procedure allows for production of nearly homogeneous preparations of all four proteins with very low LPS activity. These proteins have proven native structure and are sufficiently stable, which makes them applicable for immunological studies *in vitro* and *in vivo*.

Non-essential decorating gphoc represents a typical beta-type secondary structure. This structure has been investigated by [17] in a T4-like bacteriophage, RB49, whose gphoc has only 22% sequence identity with T4. In gphoc of RB49, beta structure also prevailed, which suggests that it might be a feature of these proteins in T4-like phages displaying gphoc. Both of these proteins contain predicted Ig-like domains that are perfectly compatible with high content of beta structure. The structure of other proteins was mixed (Table 3).

Stability studies were performed by monitoring the CD signal of protein solutions supplemented with increasing concentrations of denaturant (guanidine hydrochloride). The least stable were gp23 and Soc, having GdnHCl ½ of 1.16 M and 0.66 M, respectively. Hoc protein was more stable (GdnHCl½ of 2.03 M). Interestingly, gp24 expressed two phases of denaturation, which corresponds well with its structure and existence of two separate domains, a smaller domain A (GdnHCl½ of 0.133 M) and a larger, more stable domain B (GdnHCl½ of 2.42 M). The smaller domain of gp24 protein consists of only three beta strands and one alpha helix, and has no contacts with the larger domain. Thus we assume that out of the two domains the smaller one has lower stability. Interestingly, two essential capsid proteins, gp23 and gp24, which are known to be homologous, differ substantially in their stability *in vitro*.

The four proteins characterised in this work make up the greater part of the T4 phage capsid surface, thus being important targets for immunological studies. Although not involved directly in the processes of infection and lysis of the host cell, they may play a key role for phage survival in its environment, mediating complex interactions of the phage with external factors. Since T4 phage infects *E. coli*, its propagation may often be connected with mammalian organisms. Phage impact on human and animal bodies, which is mediated mostly by phage capsid proteins, is still poorly recognised, while it might be decisive for future phage applications: implementation of phage therapies, phage-based vaccines or drug carriers and other medical solutions.

## Materials and Methods

### Isolation of bacteriophage DNA

The bacteriophages were precipitated with PEG [29], suspended in TBS buffer (50 mM Tris-HCl, 10 mM NaCl, pH 7.0), centrifuged for 3 min at 15000 rpm, mixed with a 0.5 volume of phenol (pH = 8, water-Tris-saturated), centrifuged for 1 min at 4000× g (phenol was discarded), mixed with a 0.5 volume of chloroform, centrifuged for 1 min at 4000× g (chloroform was discarded), and dialysed for 48 h against deionised water.

### Construction of expression vectors

For gene cloning, Gateway recombination cloning technology (Invitrogen, Life Technologies Corporation) was applied. The system was based on recombinative introduction of DNA fragments into plasmids, employing two stage cloning: (i) construction of an entry clone in a non-expression vector: pDONR™221 (a pUC origin and universal M13 sequencing sites, kanamycin resistance) (Invitrogen, Life Technologies Corporation, http://tools.invitrogen.com/content/sfs/vectors/pdonr221_pdonrzeo_map.pdf) and (ii) transfer of the coding fragment to a destination expression vector: pDEST15 (Invitrogen, Life Technologies Corporation, http://tools.invitrogen.com/content/sfs/vectors/pdest15_map.pdf) for production of the recombinant proteins with N-terminal GST tags or pDEST24 (Invitrogen, Life Technologies Corporation, http://tools.invitrogen.com/content/sfs/vectors/pdest24_map.pdf) for production of the recombinant proteins with C-terminal GST tags. The procedures were done according to the Gateway technology manual.

The cloned genes were: *23*, *24*, *hoc*, *soc*. Proteins 23 and 24 undergo proteolytic modification during phage head maturation; thus their final forms are normally deprived antigenic epitopes present in their premature forms: 65 N-terminal amino acids (a.a.) of gp23 and 10 N-terminal a.a. of gp24. For immunological studies, these genes were cloned in their processed forms. The sequence coding for a protease-recognition site (AcTev protease, Invitrogen, Life Technologies Corporation) and recombination site were fused to the gene, allowing for protein tag removal. The proteolytic site was introduced between the genes and the affinity tags. Polymerase chain reaction (PCR) was performed in two steps: in the first using a template of T4 total DNA and in the second products of PCR I. Only *soc* gene amplification for cloning to pDEST24 was completed in one step PCR. The sequences of forward and reverse primers used to amplify each of the genes are listed in Table 4. Final PCR products were introduced into the pDONR221 using BP Clonase™ II Enzyme Mix (Invitrogen, Life Technologies Corporation) according to the manufacturer's instructions. All constructions were verified by automated Sanger sequencing with standard M13 primers using a 3730 DNA Analyzer, Applied Biosystems, Hitachi, DNA Sequencing KitBig Dye™ Terminator Cycle Sequencing version 1.1 (Oligo, Institute of Biochemistry and Biophysics, Warsaw, Poland). The proper clones were used for recombination with the destination vector pDEST15 (Invitrogen, Life Technologies Corporation) or pDEST24 (Invitrogen, Life Technologies Corporation) in the LR reaction according to the manufacturer's instructions.

**Table 4:** The sequences of PCR primers used for bacteriophage gene cloning.

Primer names	PCR I	PCR II
23/pDEST15/F	(5′)GAAAACCTGTATTTTCAGGGCAGCAGCAGCATGGCTGAAATCGGTGGTG(3′)	(5′)GGGGACAAGTTTGTACAAAAAAGCAGGCTCCGAAAACCTGTATTTTCAGGGC(3′)
23/pDEST15/R	(5′)GGGGACCACTTTGTACAAGAAAGCTGGGTCCTAGATACCTTTAACATATAC(3′)	(5′)GGGGACCACTTTGTACAAGAAAGCTGGGTCCTAGATACCTTTAACATATAC(3′)
24/pDEST15/F	(5′)GAAAACCTGTATTTTCAGGGCAGCAGCAGCATGTCAACCACAACGAATAG(3′)	(5′)GGGGACAAGTTTGTACAAAAAAGCAGGCTCCGAAAACCTGTATTTTCAGGGC(3′)
24/pDEST15/R	(5′)GGGGACCACTTTGTACAAGAAAGCTGGGTCCTATTCATCAATGATAATTTTTGG(3′)	(5′)GGGGACCACTTTGTACAAGAAAGCTGGGTCCTATTCATCAATGATAATTTTTGG(3′)
Hoc/pDEST15/F	(5′)GAAAACCTGTATTTTCAGGGCAGCAGCAGCATGACTTTTACAGTTG(3′)	(5′)GGGGACAAGTTTGTACAAAAAAGCAGGCTCCGAAAACCTGTATTTTCAGGGC(3′)
Hoc/pDEST15/R	(5′)GGGGACCACTTTGTACAAGAAAGCTGGGTCCTATGGATAGGTATAGATGATACC(3′)	(5′)GGGGACCACTTTGTACAAGAAAGCTGGGTCCTATGGATAGGTATAGATGATACC(3′)
Soc/pDEST15/F	(5′)GAAAACCTGTATTTTCAGGGCAGCAGCAGCATGGCTAGTACTCGCGG(3′)	(5′)GGGGACAAGTTTGTACAAAAAAGCAGGCTCCGAAAACCTGTATTTTCAGGGC(3′)
Soc/pDEST15/R	(5′)GGGGACCACTTTGTACAAGAAAGCTGGGTCCTAACCAGTTACTTTCCAC(3′)	(5′)GGGGACCACTTTGTACAAGAAAGCTGGGTCCTAACCAGTTACTTTCCAC(3′)
Soc/pDEST24/F	(5′)GGCAAAGTTTGTACAAAAAAGCAGGCTGAAGGAGATATACATATGGCTAGTACTCGCGGTTATG(3′)	-
Soc/pDEST24/R	(5′)GAACCACTTTGTACAAGAAAGCTGGGTCGCCCTGAAAATACAGGTTTTCGCTGCTGCTACCAGTTACTTTCCACAAATC(3′)	-
31/pET28b/F	(5′)AACCATGGCTGAAGTACAACAGCTACC(3′)	-
31/pET28b/R	(5′)AAGAATTCATATTACTATTTATATCAC(3′)	-

F: Forward Primer; R: Reverse primer.

### Protein expression

The following bacterial strains were used in this work: B834(DE3) F^−^ ompT hsdS_B_(r_B_^−^ m_B_^−^) gal dcm met (DE3) (EMD, Europe), Rosetta 2(DE3)Singles F^−^ ompT hsdS_B_(r_B_^−^ m_B_^−^) gal dcm (DE3) pRARE2 (Cam^R^) (Novagen-Merck Biosciences, Poland), ArcticExpress (DE3)RIL F^–^ ompT hsdS(rB^–^ mB^–^) dcm^+^ Tet^r^ gal λ(DE3) endA Hte [cpn10cpn60 Gent^r^] [argU ileY leuW Str^r^] (Agilent Technologies, Perlan Technologies, USA), BL21 (DE3)pLysS F^–^, ompT, hsdS_B_ (r_B_^–^, m_B_^–^), dcm, gal, λ(DE3)), pLysS, Cm^r^ (Promega Corporation, USA). All the strains were checked for their ability to express the recombinant T4 proteins. Since no substantial differences in protein yield were found, further work was done with B834(DE3) strain and with ArcticExpress (DE3)RIL as a donor of the specific chaperones.

Chaperones and chaperone sets used: Cpn10+ Cpn60 of *Oleispira antarctica* (in the ArcticExpress (DE3)RIL, gentamicin resistance, Agilent Technologies, Perlan Technologies, USA), groES + groEL of *E. coli* (from pGRO7 vector, chloramphenicol resistance, L-arabinose induction, TaKaRa Bio INC., Europe), dbaK + dnaJ + grpE of *E. coli* (from pKJE7 vector, chloramphenicol resistance, L-arabinose induction, TaKaRa Bio INC. Europe), TF of *E. coli* (from pTf16 vector, chloramphenicol resistance, L-arabinose induction, TaKaRa Bio INC., Europe), and gp31 of T4 phage. Gp31 is a specific co-chaperonin required for gp23 folding, which is normally expressed from T4 phage genome during phage infection of *E. coli*. This chaperone was expressed from pG31t expression vector obtained by cloning of gene *31* PCR-amplified (for primers see Table 4) to pET28c plasmid under the control of T7 promoter and of the vector ribosome-binding site, and subsequent recloning of the BglII – SspI fragment containing T7 polymerase promoter, the target gene and transcription terminator to the plasmid pLysS (Novagen) by the restriction sites BamHI and EcoRV (using compatible cohesive ends produced by BglII and BamHI enzymes and the blunt ends produced by EcoRV and SspI). Due to pACYC origin of replication, pLysS is compatible with the majority of expression vectors used for *E. coli* protein expression, and allows for overproduction of soluble gp31 (up to 30% of total cell protein), which may be somewhat decreased if co-expression with another highly produced protein is performed.

Expression conditions were individually optimized for protein yield and solubility. Expression strains were cultured with intensive aeration in LB high salts (10 g/l of NaCl) culture medium (Sigma-Aldrich, Europe or AppliChem, Europe) in the volume 15–25 litre, with appropriate selection antibiotics at 37°C (ArcticExpress strain at 30°C) and induced with IPTG (0.2 mM) and/or L-arabinose (3 mM) in the exponential growth phase as determined by OD_600_ measurements (usually at OD_600_  = 0.8). Further expression was conducted overnight at 25°C (gp23, gp24, gphoc in B834) or at 20°C (gpsoc in B834) or for 48 hours at 10°C (gp23 in ArcticExpress). Bacteria were harvested by centrifugation (6000 rpm, 5 min) and used in further procedures.

### Purification of soluble proteins in native conditions

Harvested bacteria were suspended in phosphate buffer (50 mM Na_2_HPO_4_, 300 mM NaCl, pH 7.5), PMSF was added up to 1 mM and the suspension was incubated on ice for 0.5 h. The lysis was done by freeze-thawing (−80°C) after incubation with lysozyme (50 mg/ml) for 1 hour on ice. Optimization of the procedure revealed that gp23, gp24, and gpsoc were stable in prolonged freezing (1–7 days applied) and gphoc was treated with lysozyme immediately after harvesting and then frozen only for 1 h at −80°C and immediately used for further procedures. The preparation was then supplemented with Mg^2+^ (to 0.25 mM), DNase (5 µg/ml), RNase (10 µg/ml) and incubated on ice for 1 hour. Fractions were separated by double centrifugation (22 000 g, 40 min, 4°C). Supernatant (soluble fraction) was filtered through 0.45 µm PVDF filters and incubated overnight at 10°C with 5–10 ml of glutathione sorbent slurry (Glutathione Sepharose 4B, GE Healthcare Life Sciences, Europe). Then the slurry was washed with 4 litres of phosphate buffer (50 mM Na_2_HPO_4_, 300 mM NaCl, pH 7.5) in a chromatography column. The recombinant proteins were cleaved “on the resin” by AcTev protease (5 U/ml) (Invitrogen, Life Technologies Corporation) at 10°C. The process was monitored by SDS-PAGE and terminated after 4–8 days, depending on individual results. Protein preparations were released from columns and concentrated on Vivaspin centrifuge concentrators (Sartorius, Poland) to 2–10 mg/ml.

Proteins expressed in *E. coli* usually contain a significant amount of endotoxins. LPS activity in protein preparations obtained after affinity chromatography usually exceeds 10 000 units per ml (as estimated in our work by Limulus Amebocyte Assay, Lonza, Poland). The first step of LPS removal was done with EndoTrap™ Blue (Hyglos GmbH, Germany): 50 µl of the resin per ml of a protein sample was incubated at room temperature for 1 h, centrifuged (1200 g, 5 min), the resin was removed and the cycle was repeated 3 times. Samples were dialysed against S-buffer (50 mM Na_2_HPO_4_, 150 mM NaCl, pH 8.0) and separated by gel filtration FPLC (Fast Protein Liquid Chromatography) on a Superdex 75 10/300 GL column (GE Healthcare Life Sciences, Europe) with flow rate 0.5 ml/min, at room temperature; the eluent was 50 mM phosphate buffer with 0.15 M NaCl, pH 8.0. Separation was monitored and time of elution was individually fitted. Fractions were monitored chromatographically and by SDS-PAGE gels. The final step of LPS removal was performed with EndoTrap™ Blue (Hyglos GmbH, Germany): 50 µl of the resin per mL of a protein sample was incubated at room temperature for 1 h, centrifuged (1200 g, 5 min), then resin was removed and the cycle repeated 3 times. The sample was dialysed against PBS, and filtered with 0.22 µm PVDF filters (Millipore, Europe). Protein concentration was determined by Lowry chromogenic method (Fermentas International INC.)

### LPS content determination

Endotoxin level of the purified proteins was assessed using the Limulus Amebocyte Lysate test (QLC – 1000 Chromogenic End – point LAL, Lonza). 50 µl of examined samples were transferred into endotoxin free tubes. All preparations had a negative control (i.e. apyrogenic LAL water) and were prepared in duplicate. Samples along with 4 standards were kept at 37°C for 5 min. 50 µl LAL lysate was added and incubated for 10 min at 37°C. Next 100 µl of the substrate was added, mixed gently and incubated for 8 min at 37°C. The reaction was terminated by adding the stopping reagent and mixed. 200 µl of samples were transferred to a microplate and the absorbance at 405–410 nm was measured within a 30-min period.

### Circular dichroism (CD)

CD spectra were recorded in the wavelength range 190–365 nm in PBS buffer at 21°C on a Jasco J-715 spectropolarimeter. Spectra were acquired at micromolar protein concentrations using a 1 mm cuvette with a slit width set to 5 nm and a response time of 2 s. Three independent measurements were done.

Stability studies monitored by circular dichroism: studied proteins (in PBS) were denatured with increasing concentrations of guanidinium chloride (GdnHCl) and incubated for 12 hours at room temperature; subsequently, their CD spectra were recorded as described above. The stability of the proteins was expressed as the concentration of GdnHCl required to make half of the protein denatured (GdnHCl_1/2_). The apparent free-energy change in the absence of GdmCl (deltaG) was determined by fitting CD signal changes at a particular concentration of GdmCl to the equation given by Santoro and Bolen [30].

Secondary structure content determination: Acquired CD data were analysed using K2D3 software (www.ogic.ca/projects/k2d3/) to derive secondary structure content and compare it with the predicted one based on primary protein structure prediction using PREDATOR [31], [32].

### Dynamic Light Scattering

Hydrodynamic diameter of each purified protein sample was measured in multiple repeats (5 measurements, 15 runs), at least in two repeats of independent protein preparations, by Zetasizer Nano and analysed with associated software (Zetasizer Nano, Malvern, in the NeoLek Laboratory, Wroclaw, Poland). Representative readouts were presented.
